# Synthesis, Spectroscopic and Theoretical Studies of New Dimeric Quaternary Alkylammonium Conjugates of Sterols

**DOI:** 10.3390/molecules19079419

**Published:** 2014-07-03

**Authors:** Bogumił Brycki, Hanna Koenig, Iwona Kowalczyk, Tomasz Pospieszny

**Affiliations:** Laboratory of Microbiocide Chemistry, Faculty of Chemistry, Adam Mickiewicz University, Grunwaldzka 6, Poznań 60-780, Poland; E-Mails: koenig@amu.edu.pl (H.K.); iwkow@amu.edu.pl (I.K.)

**Keywords:** sterols, dimeric quaternary alkylammonium salt, conjugates, prediction of activity spectra for substances, quantum chemical calculations

## Abstract

New dimeric quaternary alkylammonium conjugates of sterols were obtained by two step reactions of ergosterol, cholesterol and cholestanol with bromoacetic acid bromide, followed by bimolecular nucleophilic substitution with *N*, *N*, *N'*, *N'*-tetramethyl-1,3-propanediamine, *N*, *N*, *N'*, *N''*, *N''*-pentamethyldiethylenetriamine and 3,3'-iminobis-(*N*, *N*-dimethylpropylamine). The product structures were conﬁrmed by spectral (^1^ H-NMR, ^13^ C-NMR, FT-IR) analysis, mass spectrometry (ESI-MS) and PM5 semiempirical methods. Additionally *in silico* studies have been conducted for the synthesized compounds on the basis of Prediction of Activity Spectra for Substances (PASS).

## 1. Introduction

Steroids are very important natural products which play a crucial role in living organisms. This class of compounds comprises hormones, bile acids, steroidal saponins, cardioactive glycosides, steroid alkaloids and sterols [1,2,3]. One of the most important group among steroids are sterols, which can be divided into three groups: (1) zoosterols—the animal sterols (e.g., cholesterol, coprosterol); (2) phytosterols—the plant or algae sterols (e.g., sitosterol, stigmasterol); and (3) mycosterols—the sterols found in fungi (e.g., ergosterol, fungisterol) [4,5,6]. These compounds are crystalline secondary alcohols. Depending on the orientation of the condensed A and B rings of the steroid skeleton we can can distinguished between normal and allo sterols. In the first of them the A/B rings have a *cis* orientation, while the second have in *trans* geometry. Furthermore, all representative sterols have a 3β-hydroxyl group in ring A, endocyclic double bonds, usually located in the C(5)=C(6) position and side chains which various degrees of unsaturation and branching [7,8].

**Scheme 1:**
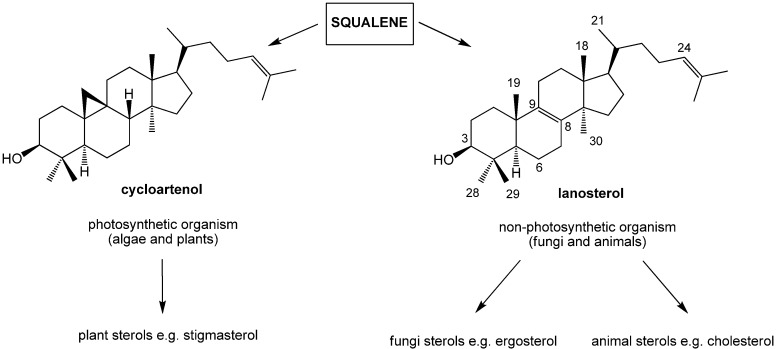
The biochemical transformations of cycloartenol and lanosterol to sterols.

In fungi and animal cells, lanosterol originated from squalene is converted into ergosterol and cholesterol. On the other hand, cycloartenol serves a similar function in algae and plant cells (Scheme 1) [2,3,4,5,6,7,8,9].

**Scheme 2:**
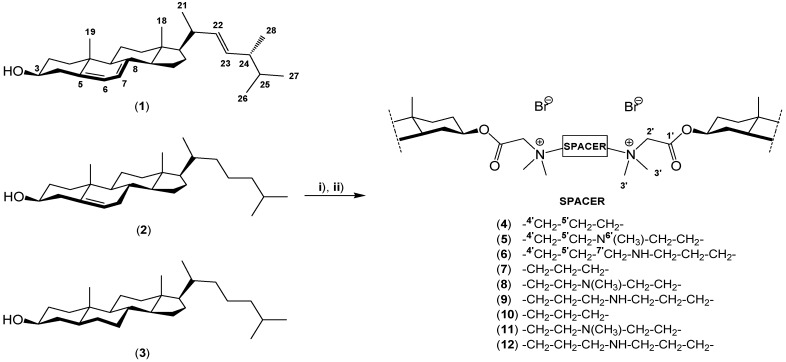
Synthesis of dimeric quaternary alkylammonium conjugates **4**–**12** of sterols **1**–**3**.

Among sterols, three of them are ubiquitous, *i.e*., ergosterol (**1**), cholesterol (**2**) and cholestanol (**3**), which is a metabolite of cholesterol (Scheme 2). Ergosterol is a vital sterol for fungal survival. It serves two purposes: a bulk membrane function and a sparking function [1,2,7,10]. Since ergosterol is part of the fungal cell membrane, fulfilling the same function as cholesterol (**2**) in animal cells, it is a target for antifungal drugs. Furthermore ergosterol is also a biological precursor to vitamin D_2_ [8,9,11,12]. 

An extremely important and well-known sterol is cholesterol. This sterol is an important component of mammalian cell membranes as in the ester form it stabilizes and stiffens protein–lipid membranes. Cholesterol is also present in significant concentrations in the brain and nervous tissue [13,14]. This animal sterol is the biosynthetic precursor of the steroid hormones, bile acids, vitamin D and lipoproteins [15,16,17,18]. On the other hand, gemini alkylammonium salts represent a new class of dimeric surfactants made up of two identical or different amphiphilic moieties having a monomeric quaternary alkylammonium salt connected by a linker (spacer) group structure [19,20]. The linker may be hydrophobic (aliphatic or aromatic) or hydrophilic (polyether, hydroxyalkyl). It can be rigid (stilbene) or flexible (a polymethylene chain). The gemini molecules have a neutral charge which is associated with the positive charge neutralization by negative ions such as e.g., halide anions [19,20,21,22,23,24]. The gemini alkylammonium salts show unique micelle-forming and surface-adsorbing properties in aqueous solution. The critical micelle concentration (CMC) for gemini surfactants is usually two orders lower than for corresponding monomeric surfactants [19,20,24,25,26,27,28,29]. The gemini alkylammonium compounds show also a very good antimicrobial activity against bacteria, viruses, molds and yeasts [30,31]. In some cases, the minimal inhibitory concentrations (MIC) of the discussed compounds are even three orders of magnitude lower in comparison to non-gemini connections [25,32,33]. The mechanism of biocidal activity of quaternary alkylammonium salts is based on adsorption of alkylammonium cations on the bacterial cell surface, diffusion through the cell wall and then binding and disruption of cytoplasmatic membrane. Membrane damage results in a release of potassium ions and other cytoplasmatic constituents, finally leading to cell death [25,34,35]. Frequently use of microbiocides, especially at sublethal concentrations, can lead to increasing microorganism resistance. One of the ways to overcome this serious negative side effect is the periodic application of new microbiocides with modified structures.

*Reagents and conditions*:** i)** BrCH_2_COBr, TEBA, CaH_2_, PhCH_3_; **ii**) *N,N,N',N'*-tetramethyl-1,3-propanediamine, [*N,N,N',N'',N''*-pentamethyldiethylenetriamine or 3,3'-iminobis(*N,N*,-dimethylpropylamine)], CH_3_CN, 81 °C.

*Reagents and conditions*: **i**) BrCH_2_COBr, TEBA, CaH_2_, PhCH_3_; **ii**) *N,N,N',N'*-tetramethyl-1,3-propanediamine, [*N,N,N',N'',N''*-pentamethyldiethylenetriamine or 3,3'-iminobis (*N,N*,-dimethylpropylamine)], CH_3_CN, 81 °C.

Potential pharmacological activities of the synthesized compounds have been found on the basis of computer-aided drug discovery approach with *in silico* of the successful use of the PASS approach leading to new pharmacological agents [36,37,38,39,40]. Through the use of PASS the biological activity of the obtained compounds may be analyzed. This approach can be used at the earliest stages of investigation, because only the structural formula of the compound is necessary to obtain a PASS prediction.

## 2. Results and Discussion

However, to the best of our knowledge, no work has been published on the synthesis and physicochemical properties of dimeric quaternary alkylammonium conjugates of sterols. These new conjugates were obtained by reaction of sterols (ergosterol (**1**), cholesterol (**2**), cholestanol (**3**)) with bromoacetic acid bromide to give ergosteryl 3β-bromoacetate, cholesteryl 3β-bromoacetate and dihydrocholesteryl 3β-bromoacetate [41,42]. 

The bromoacetates were the subjected to reactions with *N*,*N*, *N'*, *N'*-tetramethyl-1,3-propanediamine, *N*, *N*, *N'*, *N''*, *N''*-pentamethyldiethylenetriamine and 3,3'-iminobis(*N*, *N*-dimethylpropylamine). Reactions were carried out in acetonitrile at reflux for different times. These conditions favor bimolecular nucleophilic substitution, and optimize the reaction yields. The syntheses of dimeric conjugates **4**–**12** are shown in Scheme 2.

The structures of all synthesized compounds were determined by ^1^ H- and ^13^C-NMR, FT-IR and ESI-MS spectra. Moreover, PM5 calculations [43,44,45] were performed for all compounds. Additionally the pharmacotherapeutic potential of the synthesized compounds has been estimated on the basis of Prediction of Activity Spectra for Substances (PASS).

**Figure 1:**
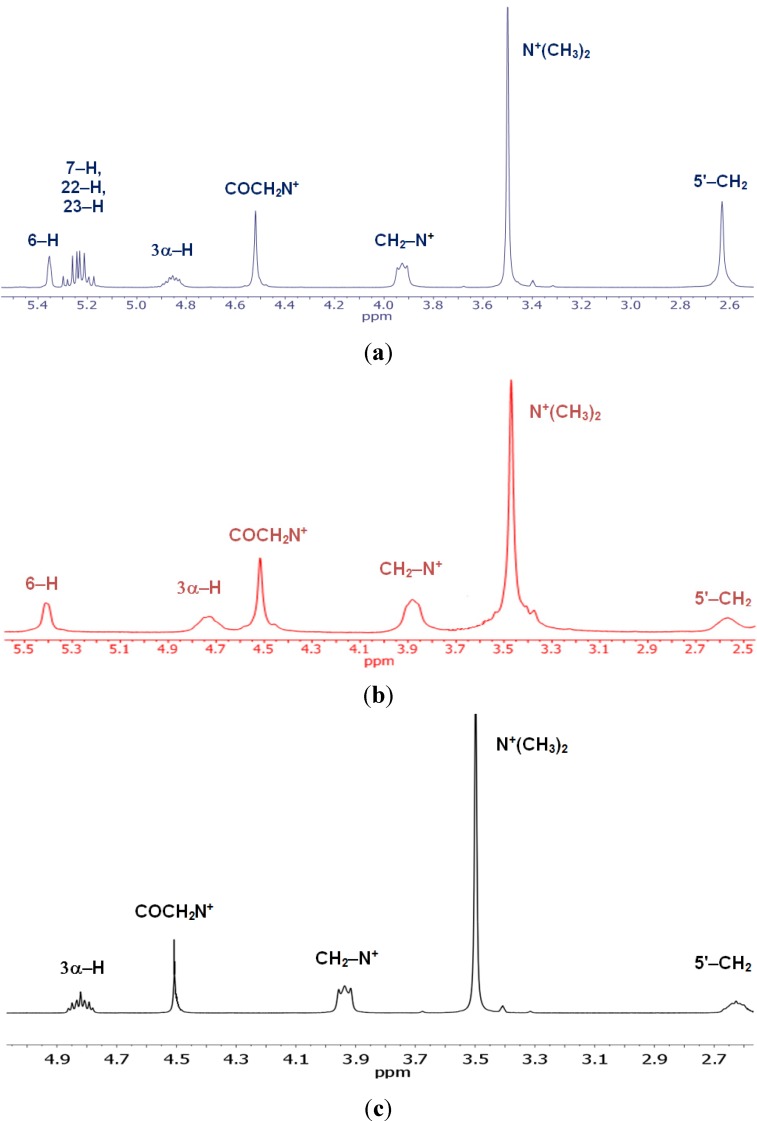
.^1^H-NMR spectra in the 5.5–2.5 ppm region showing the most characteristic signals of conjugates 4 (**a**); 7 (**b**); and 10 (**c**).

The ^1^H- and ^13^C-NMR data spectra were assigned on the basis of our previous study [41,42]. The ^1^H-NMR spectra of **4**–**12** show a signal in the range 4.65–4.48 ppm for the protons of the COCH_2_N^+^group. The signals for six methyl protons of the N^+^(CH_3_)_2_ and two methylene protons of the N^+^CH_2_occurred as singlets and triplets in the 3.58–3.46 and 4.44–3.89 ppm range, respectively (Figure 1).

The ^1^H-NMR spectra of compounds **4**–**12** show characteristic multiplets in the 4.92–4.61 ppm range assigned to the C3α–H protons of the sterol skeleton. Characteristic hydrogen singlets ranging from 0.69–0.65 ppm for **7**–**9** and **10**–**12** are assigned to CH_3_–18. Moreover, very characteristic triplets appear in the 0.84 ppm region for CH_3_–18 and CH_3_–26 as well as CH_3_–27 of the ergosterol substituted derivatives **4**–**6**. Ergosterol derivatives show multiplets in the 5.30–5.16 ppm range assigned to H–7, H–22 as well as H–23 protons. The second sets of singlets ranging from 1.03–0.82 ppm were assigned to CH_3_–19 for all compounds. The characteristic doublets of CH_3_–21 are 0.93–0.90 in dimeric conjugates. The ^1^H-NMR spectra of **7**–**12** show a doublet of doublets at 0.87–0.86 ppm for the protons of the C(26) and C(27) methyl groups. For compounds **4**–**6** a doublet appears in the 1.04 ppm assigned to CH_3_–28. The ^1^H-NMR spectra of compounds **4**, **7**, **10** show triplets in the 2.64–2.54 ppm range assigned to the N–CH_2_– protons of the propylene spacer. On the other hand, methylene protons of N(CH_3_)–CH_2_– for **5**, **8**, **11** and NH–CH_2_– for **6**, **9**, **12** appear in the range of 3.13–3.10 and 2.95–2.90 ppm, respectively. The signals for three methyl protons of the N–CH_3_ appear as singlets in the 2.38–2.35 ppm range.

The ^13^C-NMR spectra of compounds **4**–**12** are summarized in Table 1. The spectra show characteristic signals at 15.79–15.63 ppm (compounds **4**–**6**) and 12.12–11.65 ppm (compounds **7**–**12**), which are assigned to CH3–18. In a series of **10**–**12** derivatives the signals from the CH3–18 group carbon atoms from both steroid skeletons are observed (Table 2). The carbon atoms signal of the CH3–19 and CH3–21 groups appear in the 19.15–18.03 ppm and 21.13–18.50 ppm range, respectively. Furthermore, the CH3–26/27 groups are shown in the range of 22.59–22.47 ppm for **7**–**12** and 19.60–19.45 ppm for **4**–**6**. On the other hand for compounds **4**–**6** a diagnostic signal of carbon atom of CH3–28 group is observed at 17.62–17.47 ppm. Two important signals for C(1')=O and C(3)–O were present at 164.07–163.57 ppm and 77.20–76.55 ppm, respectively. The spectra of all dimeric compounds show two diagnostic signals associated with the CH_2_ atoms in N^+^–C(2')H_2_–CO and N^+^–C(4')H_2_ groups. The carbon atoms in the first group are located at 65.61–61.49 ppm and the second group at 62.24–61.48 ppm, respectively. It is noteworthy that for the –(CH_2_)_3_– linker (compounds **4**, **7**, **10**) the carbons of the CH2 groups gave signals in the 62.71–62.07 ppm range. In turn, for the linker [(CH_2_–CH_2_)_2_NCH_3_] (compounds **5**, **8**, **11**) they were at 61.91–61.49 ppm, and for the linker [(CH_2_–CH_2_–CH_2_)_2_NH] (compounds **6**, **9**, **12**) they were in 65.61–65.58 ppm. The N^+^(CH_3_)_2_ carbon atoms resonate in the 52.21–50.81 ppm range.

**Figure 2:**
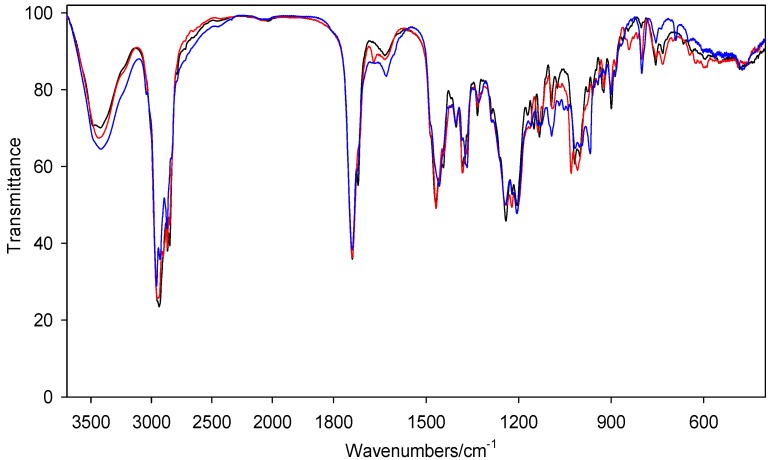
.FT-IR spectra of conjugates **6** (blue), **9** (red), **12** (black) in the 3,500–600 cm^−1^ region.

**Figure 3:**
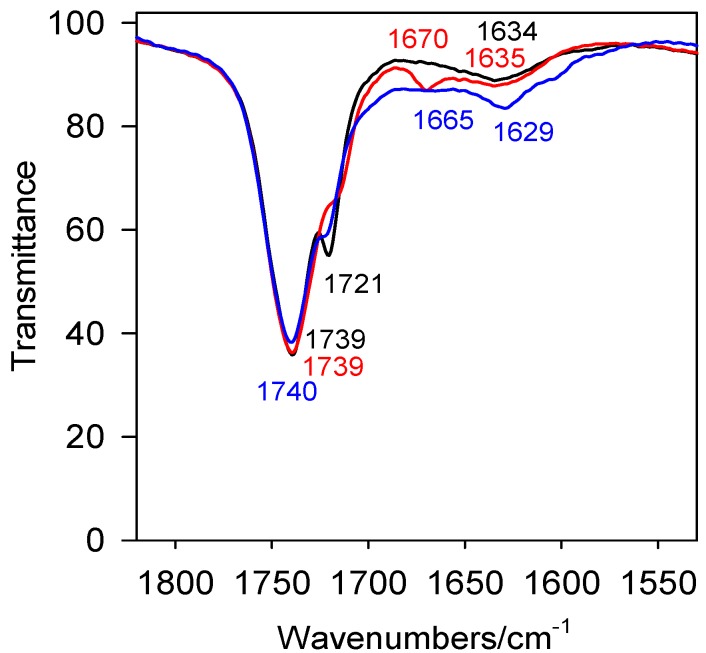
.FT-IR spectra of sterol gemini conjugates in the 1,800–1,550 cm^−^^1^region for **6** (blue), **9** (red), **12** (black).

The solid-state IR spectra of representative conjugates **6**, **9** and **12** are shown in Figure 2. The strong band in the 3,350–3,330 cm^−1^ region has been assigned to the NH group with no hydrogen bond to bromide anion. The intense bands in the 1,740–1,739 cm^−1^ region are due to the carbonyl group *ν*(C=O) stretching vibrations (Figure 3). The presence of two strong band at 1,739 and 1,721 cm^−1^ for **12** indicates the non-equivalence of the carbonyl groups. The split of the carbonyl bands suggest that both carbonyl groups in **12** are involved in different interactions in the supramolecular structure. Further coupling has little or no effect on the carbonyl group vibration. Some more strong characteristic bands in the 1,248–1,227 cm^−1^ region are present, which are assigned to the *ν*(C–O). The *ν*(C=C) stretching vibration bands of compounds **6** and **9** occur at 1,665 cm^−^^1^and 1,670 cm^−1^ respectively; for compound **12** this band is absent. The δ(N-H) deformation vibration bands are observed at 1,629 cm^−1^, 1,635 cm^−1^, 1,634 cm^−1^ for **6**, **9** as well as **12**, respectively (Figure 3).

**Table 1:** The ^13^ C-NMR chemical shifts (ppm) of compounds **4**–**12**.

Carbon	4	5	6	7	8	9	10	11	12
1'	163.70	163.89	164.07	163.57	163.75	163.95	163.59	163.87	163.99
2'	62.71	61.91	65.61	62.07	61.54	65.59	62.24	61.49	65.58
3'	51.58	52.15	50.82	51.72	52.18	50.81	51.49	52.21	50.82
4'	62.07	61.88	61.62	61.66	61.52	61.58	62.24	61.48	61.59
5'	–	56.92	–	–	55.96	–	–	54.02	–
6'	–	49.67	–	–	49.82	–	–	49.88	–
7'	–	–	57.01	–	–	56.60	–	–	54.02
3	76.77	76.56	76.55	77.19	76.67	77.13	77.15	76.97	77.20
5	139.75	139.73	139.87	138.56	138.55	138.74	–	–	–
6	123.26	123.21	123.30	123.37	138.55	123.38	–	–	–
7	118.08	118.02	118.11	–	–	–	–	–	–
8	150.47	150.45	150.55	–	–	–	–	–	–
18	15.68	15.63	15.79	11.65	11.66	11.77	11.86, 11.96	11.88, 11.99	11.92, 12.12
19	18.08	18.03	18.23	18.99	19.01	19.15	18.45	18.48	18.58
21	19.83	19.77	19.92	18.50	18.51	18.64	21.04	21.07	21.13
22	135.33	135.30	135.42	–	–	–	–	–	–
23	132.02	131.98	132.07	–	–	–	–	–	–
26/27	19.51	19.45	19.60	22.56	22.59	22.47	22.57	22.58	22.47
28	17.52	17.47	17.62	–	–	–	–	–	–

**Table 2:** Heat of formation (HOF) [kcal/mol] of sterols (**1**–**3**) and their conjugates **4**–**12**.

Compound	HOF [kcal/mol]	HOF *syn* Conformer [kcal/mol]	HOF *anti* Conformer [kcal/mol]	ΔHOF *_syn_* [kcal/mol]	ΔHOF *_anti_* [kcal/mol]
**1**	−97.1208	-	-	-	-
**2**	−140.1058	-	-	-	-
**3**	−162.7945	-	-	-	-
**4**	-	−262.2720	−255.9961	−165.1512	−158.8753
**5**	-	−250.2337	−250.1800	−153.1129	−153.0592
**6**	-	−276.4203	−257.6516	−179.2995	−160.5308
**7**	-	−345.4121	−341.1838	−205.3063	−201.0780
**8**	-	−338.9490	−332.2815	−198.8432	−192.1757
**9**	-	−358.2366	−342.9308	−218.1308	−202.8250
**10**	-	−393.0944	−386.6933	−230.2990	−223.8988
**11**	-	−380.3771	−377.8571	−217.5826	−215.0626
**12**	-	−407.2888	−388.2125	−244.4943	−225.4180

ΔHOF *_syn_* = HOF_conjugates (**4**–**12**)_ − HOF_sterols (**1**–**3**)_; ΔHOF *_anti_* = HOF_conjugates (**4-12**)_ − HOF_sterols (**1**–**3**)_.

**Figure 4:**
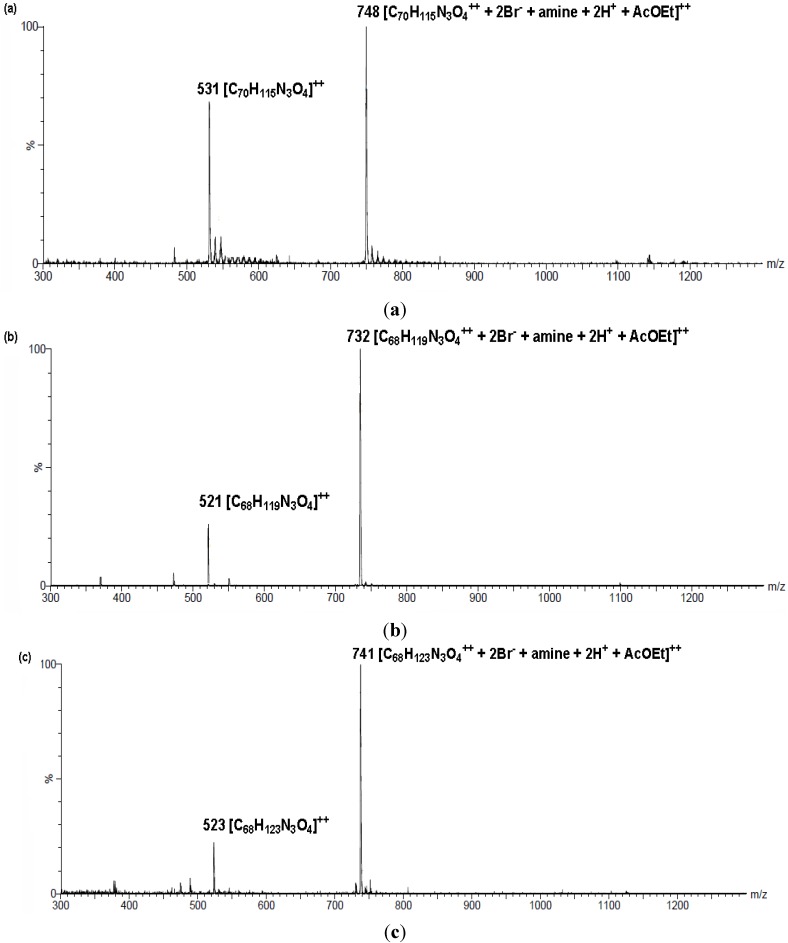
.ESI-MS spectrum of compounds **6** (**a**); **9** (**b**); **12** (**c**).

The ESI-MS spectra were recorded in methanol. The ESI-MS spectra of compounds **6**, **9** and **12** are presented in Figure 4. The ESI mass spectrometry fragmentation pathways of these compounds is poor. It is rather more diagnostic than analytical. The molecular ion [M]^+^ is not observed. Furthermore, in the spectra of these compounds, the [M]^2+^ ion peaks are observed at *m*/*z* 531 (69%), 521 (28%) as well as 523 (23%) for **6**, **9** and **12**, respectively. In the discussed compounds, the presence of [M^2+^+2Br^−^+amine+2H^+^+AcOEt]^2+^ ions (100%) was observed at *m/z* 748, 732 and 741 for **6**, **9** as well as **12**, respectively. For all other compounds (**4**, **5**, **7**, **8**, **10**, **11**) were seen ions [M]^2+^ and [M^2+^+Br^−^]^+^.

**Figure 5:**
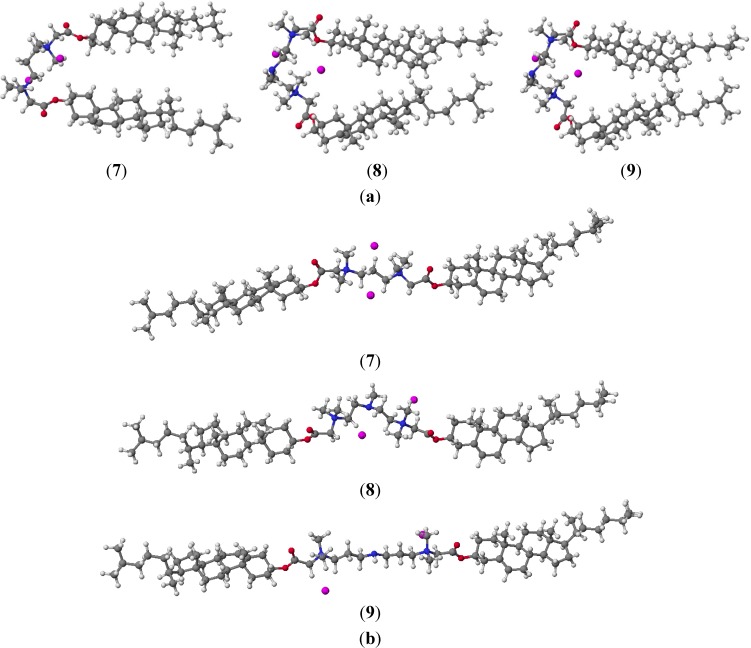
The *syn* (**a**) and *anti* (**b**) conformers of compounds **7**, **8** and **9** calculated by the PM5 method.

PM5 semiempirical calculations were performed using the WinMopac 2003 program [43,44,45]. The final heat of formation (HOF) for the sterols **1**–**3** and conjugates **4**–**12** is presented in Table 2. Representative compounds **7**, **8** and **9** are shown in Figure 5.

In ergosterol derivatives **4**–**6** and cholesterol ones **7**–**9** where double bonds are present increasing HOF values are observed. The lowest HOF value is observed for cholestanol and its derivatives **10**–**12** where there are no double bonds to stabilize the molecules and hinder their reactivity. Two low energy *syn* and *anti* conformers are possible for the dimeric-type quaternary alkylammonium conjugates of sterols as a consequence of the free rotation of bonds in the linker fragment. Semiempirical calculations on compounds **4**–**12** showed that the *syn* conformer was more stable than the *anti* conformer by 0.05–19.07 kcal/mol. The distances between the quaternary nitrogen and the anion bromide are 3.70–4.66 Å for *syn* and 3.46–3.88 Å for *anti* conformers, respectively. The distances between the quaternary nitrogen atoms and the oxygen atoms are 2.89–3.35 Å for *syn* and 2.93–3.88 Å for *anti* conformers, respectively. Compensation charges occurs only through intramolecular electrostatic interaction. In optimized structures, the anion is not engaged in hydrogen bond but in electrostatic interactions what is also confirmed by FT-IR studies in the solid phase. The variations of dihedral angles reflects the flexibility of the N^+^CH_2_COOR moiety. The PM5 calculations indicate that the conformations of all molecules are controlled by the electrostatic interactions of the positively charged nitrogen atom with the oxygen atoms of the COOR moiety and bromide anions. This is a very good confirmation of the conclusion that interactions reduce HOF.

Additionally, analyses of the biological prediction activity spectra for the new esters prepared herein are good examples of *in silico* studies of chemical compounds. We also selected the types of activity that were predicted for a potential compound with the highest probability (focal activities) (Table 3).

**Table 3:** .PA (Probability “to be Active”) values for predicted biological activity of compounds **4**–**12**.

Focal Predicted Activity (PA > 80)	Compounds								
	4	5	6	7	8	9	10	11	12
Glyceryl-ether monooxygenase inhibitor	89	89	88	92	92	91	95	95	94
Cholesterol antagonist	81	85	–	87	89	82	82	86	–
Antihypercholesterolemic	88	83	86	85	80	83	-	94	–
Acylcarnitine hydrolase inhibitor	–	-	-	85	80	-	96	-	93
Oxidoreductase inhibitor	87	86	85	-	-	-	-	-	-
Alkylacetylglycerophosphatase inhibitor	-	-	-	-	-	-	90	87	83
Alkenylglycerophosphocholine hydrolase inhibitor	-	-	-	-	-	-	88	82	80
Alcohol *O*-acetyltransferase inhibitor	91	90	90	-	-	-	-	-	-

The most important parameter is the predicted activity (PA). PA > 0.7 indicate a high probability of occurrence of a particular activity, which give a high chance to confirm it in real clinical tests. For most cases, PA > 0.7 have a structure similar to the structure of the known drug substance. In turn, 50 < PA < 70, are compounds for which the probability of confirmation of biological activity in biological research is smaller. On the other hand PA < 0.5 pertain to compounds for which the confirmation of biological activity in biological research is small, but this group connection may include new compounds structurally. We focused on the first compartment.

According to these data the most frequently predicted types of biological activity are: glyceryl-ether monooxygenase inhibitor, cholesterol antagonist as well as antihypercholesterolemic, respectively. The gemini quaternary alkylammonium conjugates **4**–**6** of ergosterol addition showed inhibition of oxidoreductase and alcohol *O*-acetyltransferase. On the other hand conjugates of **10**–**12** showed inhibition of alkylacetylglycerophosphatase and alkenylglycerophosphocholine hydrolase.

## 3. Experimental Section

### 3.1. General Information

The NMR spectra were measured with a Spectrometer NMR Varian Mercury 300 MHz (Oxford, UK), operating at 300.07 and 75.4614 for ^1^H and ^13^C, respectively. Typical conditions for the proton spectra were: pulse width 32°, acquisition time 5 s, FT size 32 K and digital resolution 0.3 Hz per point, and for the carbon spectra pulse width 60°, FT size 60 K and digital resolution 0.6 Hz per point, the number of scans varied from 1200 to 10,000 per spectrum. The ^13^C and ^1^H chemical shifts were measured in CDCl_3_ with methanol relative to an internal standard of TMS. Infrared spectra were recorded in the KBr pellets using a FT-IR Bruker IFS 66 spectrometer (Karlsruhe, Germany). The ESI (electron spray ionization) mass spectra were recorded on a Waters/Micromass (Manchester, UK) ZQ mass spectrometer equipped with a Harvard Apparatus (Saint Laurent, QC, Canada), syringe pump. The sample solutions were prepared in methanol at the concentration of approximately 10^−5^ M. The standard ESI-MS mass spectra were recorded at the cone voltage 30 V. The sterols and amines are the commercial products from Sigma-Aldrich (Saint Louis, MO, USA).

### 3.2. Synthesis: General Procedure for the Synthesis of Quaternary Ammonium C Onjugates of Sterols

Ergosteryl 3β-bromoacetate (cholesteryl 3β-bromoacetate or dihydrocholesteryl 3β-bromoacetate) (2.2 eq.) was dissolved in CH_3_CN (3 mL) under reflux. Then the appropriate amine [N,N,N',N'-tetramethyl-1,3-propanediamine, N,N,N',N'',N''-pentamethyldiethylenetriamine or 3,3'-iminobis(N,N-dimethylpropylamine)] (1.0 eq.) was added and the mixture heated under reflux for 20–70 h. The reaction was monitored by TLC (CHCl_3_/MeOH, 10:1). The precipitate formed was filtered off and crystallized from CH_3_CN/EtOH (90:1) (compounds **4**, **7**, **10**), or CH_3_CN/EtOH/CHCl_3_ (5:1:1) (compounds **5**, **6**, **8**, **9**, **11**, **12**) to give white solids.

*Trimethylene 1,3-bis(N, N'-dimethyl-N, N'-3β-acetate-ergosta-5,7,22-triene)ammonium dibromide* (**4**) white solid (80%), m.p. 190–194 °C. ^1^H-NMR: δ_H_ 5.36 (brs, 2H, 6–H), 5.30–5.17 (m, 6H, 7–H, 22–H, 23–H), 4.89–4.83 (m, 2H, 3α–H), 4.52 (s, 4H, COCH_2_N^+^), 3.93 (t, *J* = 8,0 Hz, 4H, CH_2_–N^+^), 3.50 (s, 12H, N^+^(CH_3_)_2_), 2.63 (d, *J* = 6.7 Hz, 2H, 5'–CH_2_), 1.04 (d, *J* = 6.4 Hz, 6H, CH_3_–28), 1.01 (s, 6H, CH_3_–19), 0.93 (d, *J* = 6.4 Hz, 6H, CH_3_–21), 0.84 (t, *J* = 6.1 Hz, 18H, CH_3_–18, CH_3_–26, CH_3_–27). ^13^C-NMR: δ_C_ 163.70, 150.47, 139.75, 135.33, 132.02, 123.26, 118.08, 76.77, 62.71, 62.07, 56.95, 51.58, 44.67, 42.72, 40.71, 38.78, 36.73, 36.57, 36.31, 34.86, 33.72, 32.97, 27.32, 26.30, 24.99, 21.70, 20.87, 19.83, 19.51, 18.08, 17.52, 15.68. ESI-MS (*m/z*): 1085.48 (100%) [C_67_H_108_N_2_O_4_+Br]^+^, 502.79 (15%) [C_67_H_108_N_2_O_4_]^2+^. FT-IR (KBr) ν_max_: 2,958, 1,743, 1,669, 1,459, 1,389, 1,246, 897.

*N,N,N',N',N''-pentamethyl-1,4,7-triazaheptane-1,7-bis(N,N'-3β-acetate-ergosta-5,7,22-triene)ammonium* *dibromide* (**5**) white solid (52%), m.p. 194–195 °C. ^1^H-NMR: δ_H_ 5.35 (brs, 2H, 6-H), 5.30–5.18 (m, 6H, 7–H, 22–H, 23–H), 4.92–4.77 (m, 2H, 3α–H), 4.52 (s, 4H, COCH_2_N^+^), 4.01 (brs, 4H, CH_2_–N^+^), 3.49 (s, 12H, N^+^(CH_3_)_2_), 3.12 (brs, 4H, 5'–CH_2_), 2.38 ( brs, 3H, NCH_3_), 1.04 (d, *J* = 6.4 Hz, 6H, CH_3_–28), 1.01 (s, 6H, CH_3_–19), 0.93 (d, *J* = 6.4 Hz, 6H, CH_3_–21), 0.84 (t, *J* = 6.1 Hz, 18H, CH_3_–18, CH_3_–26, CH_3_–27). ^13^C-NMR: δ_C_ 163.89, 150.45, 139.73, 135.30, 131.98, 123.21, 118.02, 76.56, 61.91, 61.88, 56.92, 52.15, 50.59, 49.67, 44.63, 42.68, 40.66, 38.74, 36.69, 36.53, 36.28, 34.82, 33.69, 32.93, 27.28, 26.26, 24.95, 21.66, 20.81, 19.77, 19.45, 18.03, 17.47, 15.63. ESI-MS (*m/z*): 1128.55 (35%) [C_69_H_113_N_3_O_4_+Br]^+^, 524.32 (100%) [C_69_H_113_N_3_O_4_]^2+^. FT-IR (KBr) ν_max_: 2,955, 1,748, 1,664, 1,454, 1,389, 1,248, 894.

*N,N,N',N'-tetramethyl-1,5,9-triazanonane-1,9-bis(N,N'-3β-acetate-ergosta-5,7,22-triene)ammonium dibromide* (**6**) white solid (93%), m.p. 195–196 °C. ^1^H-NMR: δ_H_ 5.35 (brs, 2H, 6–H), 5.28–5.16 (m, 6H, 7–H, 22–H, 23–H), 4.91–4.77 (m, 2H, 3α–H), 4.65 (s, 4H, COCH_2_N^+^), 4.44 (brs, 4H, CH_2_–N^+^), 3.58 (s, 12H, N^+^(CH_3_)_2_), 2.95 (brs, 4H, 7'–CH_2_), 1.04 (d, *J* = 6.4 Hz, 6H, CH_3_–28), 1.00 (s, 6H, CH_3_–19), 0.93 (d, *J* = 6.4 Hz, 6H, CH_3_–21), 0.84 (t, *J* = 6.1 Hz, 18H, CH_3_–18, CH_3_–26, CH_3_–27). ^13^C-NMR: δ_C_ 164.07, 150.55, 139.87, 135.42, 132.07, 123.30, 118.11, 76.55, 65.61, 61.62, 57.01, 51.34, 50.82, 44.74, 42.78, 40.78, 38.85, 36.81, 36.65, 36.46, 36.39, 34.93, 33.83, 33.04, 27.83, 27.43, 26.45, 26.39, 25.13, 25.04, 21.77, 21.16, 20.96, 19.92, 19.60, 18.23, 17.62, 15.79. ESI-MS (*m/z*): 748.49 (100%) [C_70_H_115_N_3_O_4_+2Br+amine+2H+AcOEt]^2+^, 530.94 (70%) [C_70_H_115_N_3_O_4_]^2+^. FT-IR (KBr) ν_max_: 2,957, 1,740, 1,665, 1,629, 1,457, 1,367, 1,242, 899.

*Trimethylene 1,3-bis(N,N'-dimethyl-N,N'-3β-acetate-cholest-5-ene)ammonium dibromide* (**7**) white solid (88%), m.p. 196–198 °C. ^1^H-NMR: δ_H_ 5.41 (brs, 2H, 6–H), 4.75–4.69 (m, 2H, 3α–H), 4.52 (s, 4H, COCH_2_N^+^), 3.89 (t, *J* = 6.6 Hz, 4H, CH_2_–N^+^), 3.47 (s, 12H, N^+^(CH_3_)_2_), 2.57–2.54 (m, 2H, 5'–CH_2_), 1.02 (s, 6H, CH_3_–19), 0.92 (d, *J* = 6.4 Hz, 6H, CH_3_–21), 0.87 (dd, *J* = 6.6, 1.8 Hz, 12H, CH_3_–26, 27), 0.69 (s, 6H, CH_3_–18). ^13^C-NMR: δ_C_ 163.57, 138.56, 123.37, 77.19, 62.07, 61.66, 56.51, 55.97, 51.72, 49.83, 44.68, 42.13, 39.51, 39.31, 37.56, 36.65, 36.34, 35.99, 35.59, 31.71, 31.61, 28.01, 27.80, 27.33, 24.07, 23.64, 22.56, 22.30, 20.85, 18.99, 18.50, 11.65. ESI-MS (*m/z*): 1065.49 (25%) [C_65_H_112_N_2_O_4_+Br]^+^, 492.79 (100%) [C_65_H_112_N_2_O_4_]^2+^. FT-IR (KBr) ν_max_: 2,953, 1,737, 1,665, 1,462, 1,385, 1,240, 886.

*N,N,N',N',N''-pentamethyl-1,4,7-triazaheptane-1,7-bis(N,N'-3β-acetate-cholest-5-ene)ammonium dibromide* (**8**) white solid (94%), m.p. 189–191 °C. ^1^H-NMR: δ_H_ 5.41 (d, *J* = 5.1 Hz, 2H, 6–H), 4.76–4.67 (m, 2H, 3α–H), 4.48 (s, 4H, COCH_2_N^+^), 3.96 (t, *J* = 6.4 Hz, 4H, CH_2_–N^+^), 3.46 (s, 12H, N^+^(CH_3_)_2_), 3.10 (t, *J* = 6.6 Hz, 4H, 5'–CH_2_), 2.35 (brs, 3H, NCH_3_), 1.03 (s, 6H, CH_3_–19), 0.92 (d, *J* = 6.5 Hz, 6H, CH_3_–21), 0.87 (dd, *J* = 6.6, 1.8 Hz, 12H, CH_3_–26, CH_3_–27), 0.69 (s, 6H, CH_3_–18). ^13^C-NMR: δ_C_ 163.75, 138.55, 138.55, 76.67, 61.54, 61.52, 56.49, 55.96, 52.18, 50.63, 49.82 42.13, 41.82, 39.51, 39.32, 37.60, 36.65, 36.35, 35.99, 35.61, 31.71, 31.61, 28.03, 27.82, 27.37, 24.08, 23.64, 22.59, 22.33, 20.85, 19.01, 18.51, 11.66. ESI-MS (*m/z*): 1108.56 (100%) [C_67_H_117_N_3_O_4_+Br]^+^, 514.33 (18%) [C_67_H_117_N_3_O_4_]^2+^. FT-IR (KBr) ν_max_: 2,954, 1,739, 1,663, 1,464, 1,387, 1,238, 884.

*N,N,N,N',N'-tetramethyl-1,5,9-triazanonane-1,9-bis(N,N'-3β-acetate-cholest-5-ene)ammonium dibromide* (**9**) white solid (87%), m.p. 200–201 °C. ^1^H-NMR: δ_H_ 5.39 (d, *J* = 5.5 Hz 2H, 6–H) 4.72–4.61 (m, 2H, 3α–H), 4.58 (s, 4H, COCH_2_N^+^), 4.24 (t, *J* = 9.4 Hz, 4H, CH_2_–N^+^), 3.51 (s, 12H, N^+^(CH_3_)_2_), 2.91 (brs, 4H, 7'–CH_2_), 1.01 (s, 6H, CH_3_–19), 0.92 (d, *J* = 6.4 Hz, 6H, CH_3_–21), 0.87 (dd, *J* = 6.6, 1.3 Hz, 12H, CH_3_–26, CH_3_–27), 0.68 (s, 6H, CH_3_–18). ^13^C-NMR: δ_C_ 163.95, 138.74, 123.38, 77.13, 65.59, 61.58, 56.60, 56.08, 50.81, 50.06, 48.91, 42.24, 39.43, 37.71, 36.75, 36.46, 36.11, 35.71, 31.83, 31.73, 28.14, 27.93, 27.47, 24.20, 23.77, 22.73, 22.47, 20.96, 19.15, 18.64, 11.77. ESI-MS (*m/z*): 732.01 (100%) [C_68_H_119_N_3_O_4_+2Br+amine+2H+AcOEt]^2+^, 521.34 (20%) [C_68_H_119_N_3_O_4_]^2+^. FT-IR (KBr) ν_max_: 2,951. 1,739, 1,670, 1,382, 1,206, 900.

*Trimethylene 1,3-bis(N,N'-dimethyl-N,N'-3β-acetate-5β-cholestan)ammonium dibromide* (**10**) white solid (82%), m.p. 218–220 °C. ^1^H-NMR: δ_H_ 4.88–4.80 (m, 2H, 3α–H), 4.51 (s, 4H, COCH_2_N^+^), 3.94 (t, *J* = 6.6 Hz, 4H, CH_2_–N^+^), 3.50 (s, 12H, N^+^(CH_3_)_2_), 2.64-2.61 (m, 2H, 5'–CH_2_), 0.90 (d, *J* = 6.4 Hz, 6H, CH_3_–21), 0.86 (dd, *J* = 6.6, 1.8 Hz, 12H, CH_3_–26, CH_3_–27), 0.83 (s, 6H, CH_3_–19), 0.66 (s, 6H, CH_3_–18). ^13^C-NMR: δ_C_ 163.59, 77.15, 62.24, 56.23, 56.11, 54.00, 51.49, 44.49, 42.40, 39.77, 39.32, 36.45, 35.98, 35.62, 35.23, 33.48, 31.75, 28.36, 28.04, 27.82, 27.03, 24.00, 23.66, 22.57, 22.32, 21.04, 18.45, 11.96, 11.86. ESI-MS (*m/z*): 1069.53 (10%) [C_65_H_116_N_2_O_4_+Br]^+^, 494.81 (100%) [C_65_H_116_N_2_O_4_]^2+^. FT-IR (KBr) ν_max_: 2,934, 1,735, 1,464, 1,381, 1,245, 893.

*N,N,N',N',N''-pentamethyl-1,4,7-triazaheptane-1,7-bis(N,N'-3β-acetate-5β-cholestan)ammonium dibromide* (**11**) white solid (94%), m.p. 198–201 °C. ^1^H-NMR: δ_H_ 4.84–4.75 (m, 2H, 3α–H), 4.51 (s, 4H, COCH_2_N^+^), 4.05 (t, *J* = 6.6 Hz, 4H, CH_2_–N^+^), 3.52 (s, 12H, N^+^(CH_3_)_2_), 3.13 (t, *J* = 6.4 Hz, 4H, 5'–CH_2_), 2.38 (brs, 3H, NCH_3_), 0.90 (d, *J* = 6.5 Hz, 6H, CH_3_–21), 0.86 (dd, *J* = 6.6, 1.4 Hz, 12H, (CH_3_–26, CH_3_–27), 0.82 (s, 6H, CH_3_–19), 0.65 (s, 6H, CH_3_–18). ^13^C-NMR: δ_C_ 163.87, 76.97, 61.49, 56.24, 56.14, 54.02, 52.21, 50.77, 49.88, 44.51, 42.43, 41.80, 39.79, 39.34, 36.48, 36.00, 35.65, 35.27, 33.54, 31.77, 28.40, 28.06, 27.84, 27.09, 24.02, 23. 70, 23.66, 22.58, 22.33, 21.07, 18.48, 11.99, 11.88. ESI-MS (*m/z*): 1112.59 (15%) [C_67_H_121_N_3_O_4_+Br]^+^, 516.34 (100%) [C_67_H_121_N_3_O_4_]^2+^. FT-IR (KBr) ν_max_: 2,935, 1,743, 1,465, 1,379, 1,246, 895.

*N,N,N',N'-tetramethyl-1,5,9-triazanonane-1,9-bis(N,N'-3β-acetate-5β-cholestan)ammonium dibromide* (**12**) white solid (85%), m.p. 206–207 °C. ^1^H-NMR: δ_H_ 4.84–4.74 (m, 2H, 3α–H), 4.53 (s, 4H, COCH_2_N^+^), 4.23 (t, *J* = 9.4 Hz, 4H, CH_2_–N^+^), 3.51 (s, 12H, N^+^(CH_3_)_2_), 2.90 (brs, 4H, 7’–CH_2_), 0.90 (d, *J* = 6.5 Hz, 6H, CH_3_–21), 0.86 (dd, *J* = 6.6, 1.8 Hz, 12H, CH_3_–26, CH_3_–27), 0.82 (s, 6H, CH_3_–19), 0.65 (s, 6H, CH_3_–18). ^13^C-NMR: δ_C_ 163.99, 77.20, 65.58, 61.59, 56.31, 56.20, 54.07, 50.82, 44.58, 42.50, 39.87, 39.42, 36.65, 36.54, 36.08, 35.72, 35.37, 35.33, 34.12, 33.60, 31.90, 31.85, 28.45, 28.15, 27.92, 27.15, 24.11, 23.77, 22.72, 22.47, 21.13, 18.58, 12.12, 11.98. ESI-MS (*m/z*): 740.52 (100%) [C_68_H_123_N_3_O_4_+ 2Br+amine+2H+AcOEt]^2+^, 523.36 (25%) [C_68_H_123_N_3_O_4_]^2+^. FT-IR (KBr) ν_max_: 2,936, 1,739, 1,634, 1,468, 1,383, 1,241, 899.

## 4. Conclusions

Nine new dimeric quaternary alkylammonium conjugates **4**–**12** of sterols **1**–**3** were prepared by the reactions of ergosteryl 3β-bromoacetate, cholesteryl 3β-bromoacetate and dihydrocholesteryl 3β-bromoacetate with *N*, *N*, *N'*, *N'*-tetramethyl-1,3-propanediamine, *N*, *N*, *N'*, *N''*, *N''*-pentamethyldiethyl- enetriamine and 3,3'-iminobis(*N*, *N*-dimethylpropylamine) in acetonitrile. These new compounds were characterized by spectroscopic and molecular structure methods. This study clearly shows that the conformation of investigated flexible molecules in solid and gas phase are a result of electrostatic interactions rather than hydrogen bonds. The obtained conjugates may find applications in molecular recognition and in pharmacology, especially as compounds with a high antimicrobial activity.

## References

[1] Nicolaou K.C., Montagnon T. (2008). Molecules that Changed the World.

[2] Dewick P.M. (2009). Medicinal Natural Products A Biosynthetic Approach.

[3] Koskinen A.M.P. (2012). Asymmetric Synthesis of Natural Products.

[4] Fieser L.F., Fieser M. (1959). Steroids.

[5] Templeton W. (1969). An Introduction to the Chemistry of Terpenoids and Steroids.

[6] Lednicer D. (2011). Steroid Chemistry at a Glance.

[7] Parish E.J., Nes W.D. (1997). Biochemistry and Function of Sterols.

[8] Schaller H. (2003). The role of sterols in plant growth and development. Prog. Lipid Res..

[9] Jäpelt R.B., Jakobsem J. (2013). Vitamin D in plants: A review of occurrence, analysis, and biosynthesis. Front. Plant. Sci..

[10] Hanson J.R. (2008). The Chemistry of Fungi.

[11] Vance D.E., van den Bosch H. (2000). Cholesterol in the year 2000. Biochim. Biophys. Acta.

[12] Ikan R. (1991). Natural Products a Laboratory Guide.

[13] Bloch K. (1997). Sterol molecule: Structure, biosynthesis, and function. Steroids.

[14] Bloch K. (1982). 50 Years ago, the structure of cholesterol and of the bile acids. Trends Biochem. Sci..

[15] Murry K.R., Granner D.K., Mayes P.A., Rodwell V.W. (1996). Harper’s Biochemistry.

[16] Risley J.M. (2002). Cholesterol biosynthesis: Lanosterol to cholesterol. J. Chem. Educ..

[17] Hanukoglu I. (1992). Steroidogenic enzymes: Structure, function, and role in regulation of steroid hormone biosynthesis. J. Steroid Biochem. Mol. Biol..

[18] Nagrady T., Weaver D.F. (2005). Medicinal Chemistry A Molecular and Biochemical Approach.

[19] Rosen M.J., Kunjappu J.T. (2012). Surfactants and Interfacial Phenomena.

[20] Zana R., Xia J. (2004). Gemini Surfactants Synthesis,Interfacial and Solution-Phase Behavior,and Applications.

[21] Menger F.M., Keiper J.S. (2000). Gemini Surfactants. Angew. Chem. Int. Ed..

[22] Menger F.M., Littau C.A. (1991). Gemini Surfactants: Synthesis and Properties. J. Am. Chem. Soc..

[23] Hait S.K., Moulik S.P. (2002). Gemini surfactants: A distinct class of self-assembling molecules. Curr. Sci..

[24] Holmberg K., Jönsson B., Kronberg B., Lindman B. (2003). Surfactants and Polymers in Aqueous Solution.

[25] Brycki B. (2010). Gemini Alkylammonium Salts as Biodeterioration Inhibitors. Pol. J. Microbiol..

[26] Pisárčik M., Devínsky F., Lacko I. (2003). Critical micelle concentration, ionization degree and micellisation energy of cationic dimeric (gemini) surfactants in aqueous solution and in mixed micelles with anionic surfactant. Acta Facult. Pharm. Univ. Comen..

[27] Kuperkar K., Modi J., Patel K. (2012). Surface-Active Properties and Antimicrobial Study of Conventional Cationic and Synthesized Symmetrical Gemini Surfactants. J. Surfact. Deterg..

[28] Shukla D., Tyagi V.K. (2006). Cationic Gemini Surfactants: A Review. J. Oleo. Sci..

[29] Para G., Hamerska-Dudra A., Wilk K.A., Warszyński P. (2010). Surface activity of cationic surfactants, influence of molecular structure. Colloid Surf. A.

[30] Brycki B., Kowalczyk I., Koziróg A. (2011). Synthesis, Molecular Structure, Spectral Properties and Antifungal Activity of Polymethylene-α,ω-bis(*N, N*-dimethyl-*N*-dodecyloammonium bromides. Molecules.

[31] Ng C.K.L., Obando D., Widmer F., Wright L.C., Sorrell T.C., Jolliffe K.A. (2006). Correlation of Antifungal Activity with Fungal Phospholipase Inhibition Using a Series of Bisquaternary Ammonium Salts. J. Med. Chem..

[32] Laatiris A., El Achouri M., Infante M.R., Bensouda Y. (2008). Antibacterial activity, structure and CMC relationships of alkanediyl α,ω-bis(dimethylammonium bromide) surfactants. Microbiol. Res..

[33] Diz M., Manresa A., Pinazo A., Erra P., Infante M.R. (1994). Synthesis, Surface Active Properties and Antimicrobial Activity of New Bis Quaternary Ammonium Compounds. J. Chem. Soc. Perkin. Trans..

[34] Fraise A.P., Maillard J.-Y., Sattar S.A. (2013). Russell, Hugo & Ayliffe’s Principles and Practice of Disinfection, Preservation & Sterilization.

[35] Walker E.B., Paulson D.S. (2002). Quaternary Ammonium Compounds. Handbook of Topical Antimicrobials Industrial Applications in Consumer Products and Pharmaceuticals.

[36] Poroikov V.V., Druzhilovsky D., Vasilenko V. Pharma Expert Predictive Services © 2011–2013,Version 2.0. http://www.pharmaexpert.ru/PASSOnline/.

[37] Poroikov V.V., Filimonov D.A., Borodina Y.V., Lagunin A.A., Kos A. (2000). Robustness of biological activity spectra predicting by computer program PASS for noncongeneric sets of chemical compounds. J. Chem. Inf. Comput. Sci..

[38] Poroikov V.V., Filimonov D.A. (2002). How to acquire new biological activities in old compounds by computer prediction. J. Comput. Aided Mol. Des..

[39] Poroikov V.V., Filimonov D.A., Christopher H. (2005). PASS: Prediction of Biological Activity Spectra for Substances. Predictive Toxicology.

[40] Stepanchikova A.V., Lagunin A.A., Filimonov D.A., Poroikov V.V. (2003). Prediction of biological activity spectra for substances: Evaluation on the diverse sets of drug-like structures. Curr. Med. Chem..

[41] Brycki B., Koenig H., Kowalczyk I., Pospieszny T. (2013). Synthesis, spectroscopic and semiempirical studies of new quaternary alkylammonium conjugates of sterols. Molecules.

[42] Brycki B., Koenig H., Kowalczyk I., Pospieszny T. (2014). Synthesis, spectroscopic and theoretical studies of new quaternary *N*, *N*-dimethyl-3-phtalimidopropylammonium conjugates of sterols and bile acids. Molecules.

[43] (2003). CAChe 5.04 User Guide.

[44] Stewart J.J.P. (1991). Optimization of parameters for semiempirical methods. III Extension of PM3 to Be, Mg, Zn, Ga, Ge, As, Se, Cd, In, Sn, Sb, Te, Hg, Tl, Pb, and Bi. J. Comput. Chem..

[45] Stewart J.J.P. (1989). Optimization of parameters for semiempirical methods I. Method. J. Comput. Chem..

